# Potentially Toxic Element Levels in Atmospheric Particulates and Health Risk Estimation around Industrial Areas of Maros, Indonesia

**DOI:** 10.3390/toxics9120328

**Published:** 2021-12-02

**Authors:** Annisa Utami Rauf, Anwar Mallongi, Kiyoung Lee, Anwar Daud, Muhammad Hatta, Wesam Al Madhoun, Ratna Dwi Puji Astuti

**Affiliations:** 1 Department of Environmental Health, Faculty of Public Health, Hasanuddin University, Makassar 90245, Indonesia; annisautamirauf@gmail.com (A.U.R.); anwardaud66@gmail.com (A.D.); ratnadwipujiastuti@gmail.com (R.D.P.A.); 2 Department of Environmental Health Sciences, Graduate School of Public Health, Seoul National University, Seoul 08826, Korea; cleanair@snu.ac.kr; 3 Marine Science Department, Faculty of Marine Science and Fisheries, Hasanuddin University, Makassar 90245, Indonesia; hattaikl9@yahoo.com; 4 Faculty of Engineering, Gaza University, Gaza, Palestine; wsah79@gmail.com

**Keywords:** air pollution, industrial waste, risk assessment, Maros

## Abstract

Air quality deterioration is a major environmental problem in Indonesia. This study evaluated the levels and health risks of potentially toxic elements (PTEs) in Maros Regency, Indonesia. Total suspended particulate matter was collected from industrial areas for PTE (Al, Pb, Cr, Cu, Ni, As and Zn) analysis using inductively coupled plasma optical emission spectrometry (ICP-OES). Samples were collected from six critical areas in the Bantimurung region as that is where marble, cement and limestone industries are located. A calculation of the non-carcinogenic and cancer risks was performed to determine the potential health exposures in adults and children. A Monte Carlo simulation with 10,000 iterations and a sensitivity analysis was carried out to identify the risk probability and the most sensitive variable contributing to cancer risk from PTE exposure in humans. The results showed that the concentration of PTEs decreased in the order of Zn > Al > Cr > Pb > Cu > Ni > As in the wet season, and Zn > Al > Pb > As > Cr > Cu > Ni in the dry season. The hazard index (HI) value for children was 2.12, indicating a high non-carcinogenic risk for children. The total cancer risk (TCR) values in adults and children were 3.11 × 10^−5^ and 1.32 × 10^−4^, respectively, implying that both are at risk for developing cancer. The variables with the most contribution to cancer risk from As, Cr and Pb exposure in adults and children were As concentration (33.9% and 41.0%); exposure duration (ED) (34.3%) and SA (40.7%); and SA (98.7 % and 45.4%), respectively. These findings could be used as the scientific basis for public health intervention and to raise awareness of the harmful health effects of particulate bound PTEs

## 1. Introduction

Air pollution is one of the greatest environmental problems faced by developing countries. Globally, air pollution is the world’s largest environmental health risk and is responsible for 3.1% of the loss of disability-adjusted life years (DALYs) [1]. According to the Environment and Social Development Organization (ESDO), at least 200,000 people in Bangladesh could die from respiratory disease and long-term exposure to high concentrations of contaminated air [2]. Air pollution from industrial sources is associated with low birth weight and the severity of sleep disorders among the elderly in China [3]. In Indonesia, the air quality has decreased over the last two decades, and it fails to meet the World Health Organization (WHO) standard for fine particulate matter (PM_2.5_) concentration. Indonesians lose 1.2 years of life expectancy as a result of the current pollution levels [4]. Types of land use, human activities, road traffic and highly industrial activities contribute to environmental degradation, including the pollution of the air with toxic trace metals.

Potentially toxic elements (PTEs) in particulate matter from ambient air are mostly generated from vehicle fumes and industrialization [5]. In previous studies, PTEs were found to have accumulated in soil, water and the atmosphere, and exceeded the standard for environmental quality [6,7,8,9]. A study conducted by Soltani (2021) reported the presence of Cu, Co, V, Ni, Fe and Zn as the most abundant elements in total suspended particulate (TSP) matter, PM_2.5_ and PM_10_ [10]. The presence of PTEs can be estimated from the combination of geogenic and anthropogenic effects [11]. A high level of PTES was observed the contributions of Zn, As, Cd, Pb and Hg that could transport to the environments via runoff and resuspension for 39.6% (Zn), 57.9% (As), 63.8% (Cd), 52.3% (Pb) and 51.3% (Hg) of the total dust in Onsan industrial complex in Korea [12]. The composition of particulate matter also consists of Earth crust elements such as Al, Zn, Ni and Fe [6,9]. A study in Chelyabinsk mentioned that Al is the only geogenic element that is correlated with parent rocks and has the same possible sources as Co and Fe [13]. The essential metals, in certain amounts, may lead to acute exposure and non-carcinogenic effects from dermal absorption or oral inhalation [14].

Long-term exposure to PTEs in the air is harmful to human health as they contain toxic particulates in the form of heavy metals [6,9]. For example, exposure to chromium (Cr) can cause lung tumors in mice through the inhalation pathway [15], while arsenic (As) may be associated with low birth weight and infant mortality [16]. As is found in industrial dust and enters the human body via inhalation, dermal exposure and orally, and affects the metabolic processes. Pigmentation and keratosis are specific skin lesions that exhibit chronic As toxicity [17]. Low levels of As can reduce the production of erythrocytes and leukocytes, abnormal heart beat, motor dysfunction, and pricking sensation in hands and legs [17,18]. In children, the toxicological effects of Cr and Pb pose the risk of neurological diseases and typical symptoms on the skin in the form of redness, burns and itching [19]. Repeated exposure may possibly cause atopic dermatitis and skin cancer.

In Indonesia, a high level of PTEs in particulate matter was found in the metal casting and cement industry [20] in metropolitan areas and from biomass burning by local industrial sources [21]. In 2019, the diseases that were attributed to the DALYs from air particulate matter pollution in South Sulawesi were ischemic heart disease (IHD) (7.2%), stroke (10.18%), lung cancer (1.63%), diabetes mellitus (4.88%), chronic obstructive pulmonary disease (COPD) (3.24%), blindness and vision loss (1.49%), neonatal disorders (4.91%) and lower respiratory infections (LRI) (2.2%) [22]. Maros is a growth area in South Sulawesi that is home to several industrial activities, particularly limestone processing and crushed stone [23]. The number of respiratory and dermatitis cases in this area is relatively high. Moreover, the morbidity rate of residents who experience health problems and feel their ability to carry out daily activities is impacted as a result of air pollution is 15.81 per 100,000 population. In addition, cases of dermatitis increased during this period with an average of 598 individuals per year [24].

The levels of PTEs in the air around residential areas should be of major concern as the health of the human population can be greatly affected [6]. Residents living nearby industrial areas are vulnerable populations due to the higher concentrations of PTEs, and they experience more frequent exposure than those in non-industrial areas. Although numerous studies of air pollution have been carried out, little attention has been paid to PTEs in Indonesia. An analysis of the health risks from PTE exposure can provide an overview of the adverse impacts that a population may experience. The contribution of the main variable in a health risk assessment can be determined using a probabilistic Monte Carlo model [25]. By using this approach, the distribution characteristics of all variables can be shown. The complex environment and the uncertainty of this method are scientifically accurate. This approach is recommended by the United States Environmental Protection Agency (USEPA), and it considers the probability distribution for each exposure parameter [26]. The modeling results from the Monte Carlo analysis could contribute to the creation of a mitigation plan for reducing the health risk of PTEs.

This study was carried out in Maros Regency, South Sulawesi Province, Indonesia, where the main industries are karst stone processing, cement and marble. This background leads to the possibility of PTEs being released into residential areas and affecting the air quality. The aims of this study are: (1) to assess the PTE levels in total suspended particulate (TSP) matter through seasonal variation (wet and dry season); (2) to investigate the carcinogenic and non-carcinogenic risks for the local residents; and (3) to determine the most influential factors in carcinogenic risk using a Monte Carlo simulation sensitivity analysis.

## 2. Methods

### 2.1. Study Area

Maros Regency is a part of the South Sulawesi Province and is located in the north of Makassar City, Indonesia. The total area of Maros Regency is 1619 km^2^. The annual average air temperature is 29 °C and the minimum temperature is 21 °C. Maros Regency is an area with a tropical climate, due to its location at the equator, with a humidity range of 60–82%. The average annual rainfall is 347 mm/month with an average of 16 rainy days. The focus area of this study was conducted in the Bantimurung region where industrial activities for marble, cement and limestone are located.

### 2.2. Sampling Procedures

**Figure 1:**
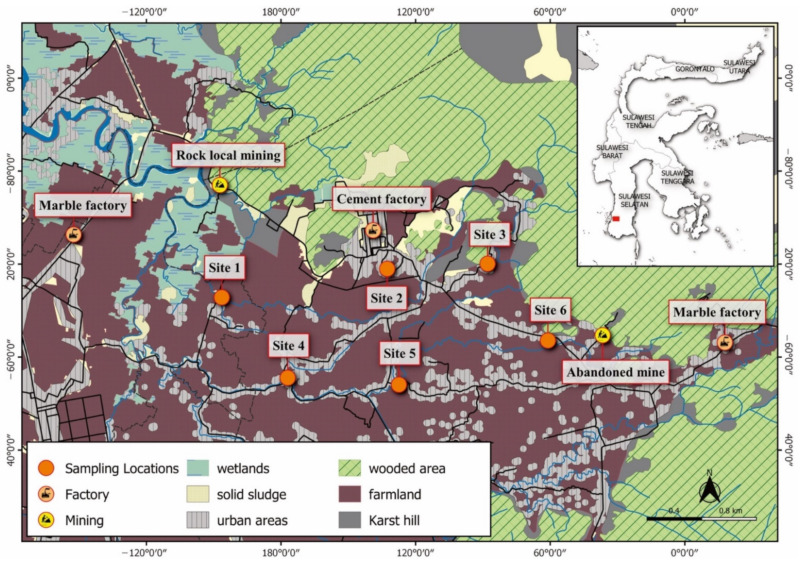
Map of the study area and sampling locations in Maros Regency.

The total suspended particulate matter was obtained with a high-volume air sampler (HVAS) (Staplex TFIA 2) and placed 1.5 m above the ground. Measurements were carried out for 24 h at six sites. Air sampling was collected for seven days in two seasons: the wet season (9 to 30 November 2020) and the dry season (23 July to August 2021). In the wet season, sampling was carried out in safe weather undisturbed by rain. To reduce the direct bias from vehicle fumes, the device was placed 200–300 m from the highway. The TSP samples of 25 mg were digested in a mixture of high-purity concentrated hydrofluoric acid, concentrated nitric acid and hydrogen peroxide (5:1:1), heating to 190 °C and cooling for 25 min. The digested samples were re-dissolved with 0.5 mL HNO_3_ diluted with ultrapure water in a 50 mL flask. The concentration of Al, As, Cr, Cu, Ni, Pb and Zn were analyzed by inductively coupled plasma optical emission spectrometry (ICP-OES). The standard solutions were determined three times to verify the precision of the instruments. The results showed good accuracy, obtained a standard deviation less than 5% and the calibration curves were linear (R^2^ greater than 0.995). The map of the study area is shown in Figure 1.

In this study, the six selected sites were chosen as they were critical residences near industrial areas. According to Figure 1, site 1 was a residence surrounded by a cement factory employee housing area and a new settlement in the west. Located around 1.4 km from the cement factory and 600 m from traditional rock mining, this site was an area traversed by heavy-duty trucks for the transportation of factory products and raw materials. This site was selected as the residents complained about the black and yellow dust appearing every morning. Sites 2 and 3 were typical village areas occupied by residents before the industrial activities began. In this area, most of the residents worked as farmers who spent their activities outdoors. Both locations were experiencing wind blows from the northwest. Site 4 was located in the south area, approximately 2.35 km from a cement factory and 2.77 km from a marble factory in the west. This area was one of the most densely populated areas where most of the residents worked as farmers. Site 5 was an area inhabited by residents in the south, where the traffic volume was relatively low. The residents mostly worked as blue-collar workers and farmers. Site 6 was also located in the south, where one marble factory was actively producing, even though the location was quite far from the local residences. In this location, there were several karst hills and most residents worked as farmers. About 700 m to the east, there was an abandoned limestone mine. No sampling was carried out in the north, northeast or northwest, as these areas were uninhabited and surrounded by Tonasa limestone formation.

### 2.3. Human Data Collection

The human data collection was carried out through individual interviews with 317 respondents (adults and children). Information on the respondents regarding the duration of residence in the study area (ED), specific body parts that were likely to be in contact with dust/skin adherence (SA), medical history and body weight (BW) were collected. The researcher and enumerators were directly involved in the data collection through door-to-door visits to residential houses. This study was approved by the Health Research Ethics Commission, Hasanuddin University (protocol No. 28920093022). Only respondents who were willing to participate, aged between 4 to 60 years (the children involved were aged between 4 and 12 years, and the adults were between 13 and 60 years), and signed informed consent were included in this study. All the respondents had been living in the study area for at least one year before the study took place. Children older than 4 years and having problems in social interaction and communication or having no consent from parents to participate were excluded. Moreover, children were accompanied by their parents when answering all questions related to this study.

### 2.4. Health Risk Assessment

The adverse effects posed by metals in environmental media can be predicted using the health risk assessment model. Air that is contaminated with particulate matter may pose a health risk due to human exposure to PTEs through inhalation and oral and skin contact [27]. The average daily dose (ADD) from trace metals (As, Al, Cr, Cu, Ni, Pb and Zn) through oral, inhalation and dermal routes can be calculated using Equations (1)–(3):

(1) $${\mathrm{ADD}}_{\mathrm{oral}}=\frac{C\;\times {\;\mathrm{Ing}}_{\mathrm{rate}}\times \;\mathrm{CF}\;\times \;\mathrm{EF}\;\times \;\mathrm{ED}}{\mathrm{BW}\;\times \;\mathrm{AT}}\;$$

(2) $${\mathrm{ADD}}_{\mathrm{inhalation}}=\frac{C\;\times {\;\mathrm{Inh}}_{\mathrm{rate}}\times \;\mathrm{EF}\;\times \;\mathrm{ED}}{\mathrm{PEF}\;\times \;\mathrm{BW}\;\times \;\mathrm{AT}}$$

(3) $${\mathrm{ADD}}_{\mathrm{dermal}}=\frac{C\;\times \;\mathrm{SA}\;\times \;\mathrm{CF}\;\times \;\mathrm{SL}\;\times \;\mathrm{EF}\;\times \;\mathrm{ABS}\;\times \;\mathrm{ED}}{\mathrm{BW}\;\times \;\mathrm{AT}}$$

A hazard quotient (HQ) was used for the non-carcinogenic health risk of a single element using Equation (4).

(4) $$\mathrm{HQ}=\frac{\mathrm{ADD}}{\mathrm{RfD}\;\mathrm{or}\;\mathrm{RfC}}$$

RfD stands for the oral reference dose in different exposure route, while RfC is defined as an estimate/reference concentration of a continuous inhalation exposure in humans. An assessment of the non-carcinogenic risk posed by multiple elements and routes was conducted using a hazard index (HI). The HI value below 1 means no carcinogenic risk; a value more than 1 indicates that the adverse effects of non-carcinogenic risk may appear. The value of HI was estimated using Equation (5).

(5) HI= Σ HQ

Carcinogenic risk (CR) is the probability of an individual suffering from carcinogenic problems in a lifetime caused by exposure to carcinogens. CR is estimated as the product of average daily dose (ADD) and cancer slope factor (CSF) over time. Total cancer risk (TCR) is the sum of CR values from individual exposure. If the value is in the range of 1 × 10^−6^–1 × 10^−4^, the risk is still low or acceptable. If the value is ≥1 × 10^−6^, the risk is unacceptable and there is the possibility of carcinogenic disease in the future. CR and TCR values can be calculated using Equations (6) and (7).

(6) CR=ADD×CSF

(7) TCR = Σ CR

The definitions, units, symbols and values used to determine the health risk assessment are presented in Table 1, while Table 2 shows the values of RfD and CSF.

### 2.5. Monte Carlo Simulation

The Monte Carlo simulation is able to show the health risk probability and shows the contribution of all variables through a sensitivity analysis. The simulation uses the random sampling of probability distributions within a predictive model, resulting in hundreds or even thousands of different options [39]. This simulation allows the estimation of uncertainty under more flexible conditions. The standard values used in a health risk analysis, such as ingestion rate, inhalation rate and cancer slope factor, can lead to uncertainty and complicated results in a risk assessment. Rather than using one-point value, a harmful risk probability can be obtained through simulation by collecting sufficient numbers of implants that share the same combination of risk factors. In this study, all the tested variables in a sensitivity analysis were related to site-specific data (interviews, questionnaires and chemical analysis), such as metal concentrations, ED, BW and SA. Meanwhile, the data from standard references were excluded (ingestion rate, inhalation rate, CF and dermal absorption factor (ABS)). The distribution of the main variables was used to find the correlation between PTE exposure and the probability of developing cancer in adults and children. The function of the selected variables in the Monte Carlo simulation is expressed in Equation (8).

(8) $$Y\;=\;h\left(X\right)\;=\;h\left(X1,\;X2,\;\ldots ,\;\mathrm{Xk}\right)$$

The variables of interest in this study were exposure duration (ED), body weight (BW), skin adherence (SA) and metal concentration (C), defined as *X* (input parameters). All of these variables were obtained directly from the questionnaires and interviews in order to get actual results from the exposure events experienced by the local residents. *Y* is the cancer risk (CR) due to inhalation, ingestion or dermal absorption of PTEs as a result of exposure. For the distribution analysis, the repetition was applied 10,000 times.

The Monte Carlo simulation was performed using the Oracle Crystal Ball (version 11.1.12.) with Microsoft Excel 2018 add-in. The output result is represented by a probability distribution histogram. Since Excel does not have the ability to run and analyze simulations and uncertainty, researchers must rely on third-party programs such as Crystal Ball that add in and expand the features of Excel. The results including statistics and probability of this study were determined using Monte Carlo simulation and Excel automatically recalculates the model [40]. Several studies have applied Monte Carlo simulation in the past few years. This simulation was employed to reduce the uncertainty and the contamination levels of toxic elements in Korean cabbage kimchi [41]. Monte Carlo can also be coupled with a dispersion model named Monte Carlo–dispersion simulation method (MCDSM) to determine the probability of odor exceedance of CH_3_SH near the landfill and produce probabilistic odor impact results [42].

## 3. Results and Discussion

### 3.1. Concentrations of TSP and PTEs

A descriptive analysis of the metals found in TSP from six locations is presented in Table 3. In this study, the TSP levels in the dry season were significantly higher than in the wet season (*p* < 0.05). Dry weather and lower humidity cause the accumulation of particulate matter in the air to be higher than the wet season. According to Table 3, the mean concentration of TSP in the study area was within the limit for 24 h, but exceeded the maximum annual standard set by Indonesia and the WHO. The mean concentration of Al in the wet and dry seasons varied from 6098.9 μg/m^3^ to 11,678.8 μg/m^3^ and from 58.6 μg/m^3^ to 1446.6 μg/m^3^, respectively. The mean concentration of As in the wet and dry seasons was 1.61 and 91.63 μg/m^3^, respectively. The Cr levels in the wet season increased almost seven times in the dry season, with an average concentration of 11.88–81.17 μg/m^3^. The Cu levels in the wet season were 4.68 μg/m^3^_,_ which drastically increased in the dry season to 78.97 μg/m^3^. The concentration range of Pb in the study area was 5.54–1968.1 μg/m^3^, with an average of 6.90 μg/m^3^ in the wet season and 746.78 μg/m^3^ in the dry season. The mean concentrations of Zn in the wet and dry seasons were 9844.5 μg/m^3^ and 23247.15 μg/m^3^, respectively. The mean concentration of Ni in the wet season was 1.81 μg/m^3^. Unlike other elements, the Ni concentration was not detected in the dry season. As one of the crustal elements, this metal is not dominant in air pollution due to its high instability, and it is found in ambient air at very low levels as a result of releases from oil and coal combustion [43].

The mean concentrations of PTEs in TSP were in decreasing order of Zn > Al > Cr > Pb > Cu > Ni > As in the wet season, and Zn > Al > Pb > As > Cr > Cu > Ni in the dry season. Overall, Zn and Al were the most abundant elements from among the metals studied in TSP. A study in Nigeria found that particulate matter was released in higher levels in the vicinity of industrial facilities during the dry and wet seasons [46]. A high concentration of Zn is emitted into the atmosphere as a result of the refining of other metals containing zinc as an impurity [47]. Zn is found to be the dominant compound in fly ash, in the form of Zn chloride (bounded to Si), and has no correlation with the seasons. As a result of this condition, Zn was found to be the major pollutant in the industrial areas. In general, Zn is bound with other elements, especially Pb, Cu and Fe, in the form of sphalerite, which can be found in high levels in the air. Based on Table 3, the mean concentration of Pb in both seasons was above the permissible limit of WHO and Indonesian standard. Pb is the most abundant element in air pollution, along with Zn [48]. The presence of Pb in the air is often caused by coal burning and transportation activities. The burning of fuel, higher dry deposition and increased vehicle activity are greater in the dry season than in the wet, causing more Pb to be distributed and accumulated in the air [49].

Continuous exposure to PTEs in residential areas can cause adverse health effects in humans, including cardiovascular diseases, genetic issues and internal organ damage [50]. In this study, toxic metals such as Pb and Cr were present in the TSP. These elements are classified as carcinogenic pollutants and harmful to humans [16]. Pb exposure in males causes a decrease in sperm quality and a possible alteration of serum levels. Young children and infants adsorb more Pb and are particularly susceptible to neurological effects [51]. The inhalation and oral intake of Cr causes Cr poisoning, pneumonia, kidney disease, gingivitis and lung cancer [16]. With dermal absorption, Cr exposure causes damage to the epidermis cells leading to dermatitis, which causes redness and burning in the skin. Furthermore, the toxicity of As compounds has been associated with metabolic processing problems [52]. As is responsible for epigenetic alterations, DNA damage and heart problems [16]. The other elements, such as Cu, Al, Zn and Ni, are some of the most abundant elements in the Earth’s crust. These types of elements are not as dangerous as toxic trace metals, but in certain amounts, can have acute effects. Dizziness and irritation of the nose, mouth and eyes can result from long-term exposure to Cu dust. Ni in high concentrations can lead to genetic determinants for nickel-induced lung toxicity and dermatitis. The accumulation of Al and Zn in the body mostly occurs in the blood, tissues and organs where repeated inhalation can lead to lung damage [53].

### 3.2. Health Exposure Assessment

#### 3.2.1. Daily Intake and Non-Carcinogenic Risk

The ADD values for adults and children are presented in Table S1. According to the ADD calculation, the major intake of PTEs in adults and children took place via ingestion, followed by inhalation and dermal route. This is a reminder that the role of the ingestion route from PTE exposure cannot be ignored. The ADD values followed the order of Pb > Zn > Al > As > Cr > Cu > Ni for inhalation and ingestion route, while the order for dermal absorption was As > Zn > Al > Pb > Cr > Cu > Ni for adults, and Zn > Al > Pb > As > Cr > Cu > Ni for children. Compared to the other elements, Zn, Pb and Al had the highest concentrations in this study, which may greatly influence the amount of intake and subsequent health effects experienced by individuals. A previous study found that the bioavailability fractions of Zn, As, Cu and Pb were more than 60% in atmospheric particulate. This implies that the dominant air pollutants and their high concentrations play an important role in the human intake risk assessment.

The HQ and HI values of PTEs from multiple pathways (inhalation, ingestion and dermal) are presented in Table 4. Based on the HQ value, adults showed the highest potential for non-carcinogenic effects from HQing of As (2.41 × 10^−1^); this result was consistent in children, where the major result was the HQing of As (9.36 × 10^−1^), followed by HQing of Pb (6.48 × 10^−1^) and HQing of Zn (3.32 × 10^−1^). These results did not exceed the threshold value of 1. However, the sum of HQ was expressed as a HI value and was used to assess the overall estimation for non-carcinogenic risk posed by more than one chemical. The HI values for adults and children were 0.64 and 2.12, respectively. These results indicate that children have a higher possibility of adverse health effects from PTE exposure than adults [38,54], while the non-carcinogenic risk from PTE exposure in adults is negligible. The high concentration of metals influenced the daily intake and hazard index values in humans. Children and infants are a vulnerable population and have a greater risk of experiencing health problems due to PTE exposure from the atmosphere than adults [6,55]. This is because the amount of air inhalation and other intake by children is twice that of adults (considering the intake per weight unit), and the fact that their lung function and immune systems are not yet fully developed [56].

#### 3.2.2. Carcinogenic Risk

The carcinogenic health risks to adults and children were determined based on the exposure to As, Pb and Cr, which are the carcinogenic pollutants. The CR values for inhalation, ingestion and dermal exposure are listed in Table 5. The CR value decreased in the order of ingestion > dermal > inhalation, with ingestion posing the most significant risk. The carcinogenic risks of Pb and Cr from ingestion were slightly above the acceptable level range (1.0 × 10^−4^–1.0 × 10^−6^), both in children and in adults. In addition, the CR value of As through combined pathways was negligible as their values were less than the acceptable value. However, exposure to As, even in small amounts, is very dangerous in the long term because of its strong toxicity.

The TCR was calculated as the sum of all CRs for all combined routes. The TCR values for adults (3.11 × 10^−5^) was lower than for children (1.32 × 10^−4^). These values exceeded the maximum acceptable risk [38] and indicated a higher probability of cancer occurring in children from PTE exposure. This is of major concern and an important finding, as the residents who live in these industrial areas are in contact with and exposed to PTEs simultaneously. Long-term exposure to PTEs can cause DNA damage and neurological problems in children [57,58]. Moreover, elevated levels of carcinogenic elements are associated with breast cancer, liver dysfunction, central nervous system damage and lung cancer [16].

### 3.3. Monte Carlo Simulation

The distribution frequencies of the cancer risks are shown in Figure S1. The potential exposure to As was slightly below the cancer risk threshold limit and was negligible for adults and children, whereas the cancer risk values of Pb and Cr revealed the high potential for cancer effects to be experienced by the local residents. The high cancer risks were confirmed by the mean, 5% percentile and 95% percentile cumulative probabilities using the Monte Carlo simulation. We recommend the periodic monitoring of the concentration and distribution of pollutants in air environments. It is necessary to consider the local level regulations, specifically environmental safety regulations, around industrial areas.

The most influential variables for cancer risk of As in adults and children were As concentration (33.9% and 41.0%). For cancer risk of Cr, the most influential variables in adults and children were ED (34.3%) and SA (40.7%), respectively. Moreover, SA was the most important variable in the cancer risk of Pb, both in adults (98.7%) and children (45.4%). Body weight (BW) showed negative results on the sensitivity analysis for As in children (−20.4%), Cr in adult (−32.3%) and Pb in adult and children (−0.5% and −17.9%), indicating no significant effect on the cancer risk. The high contribution of SA to the development of cancer risk from exposure to Pb and Cr indicated that exposure to these metals is very dangerous and has potential adverse health effects. It is recommended that residents use more skin protection, especially for those who spend more time outside.

**Figure 2:**
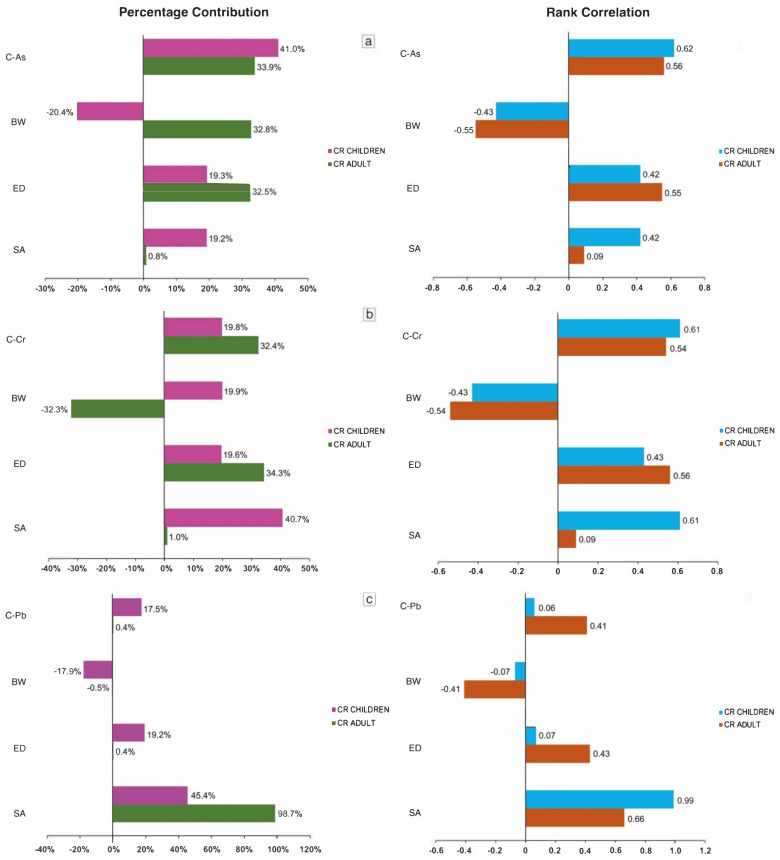
Sensitivity analysis results for cancer risk estimation in adults and children: (**a**) cancer risk of As; (**b**) cancer risk of Cr; and (**c**) cancer risk of Pb.

Figure 2 shows the correlation results for the key variables that contribute to cancer risk. The CR values of As have a strong correlation with As concentration both in adults (0.56) and children (0.62), followed by ED in adults (0.55) and children (0.42). The CR values of Cr were found to have a significant correlation with Cr concentration for adults (0.54) and children (0.61). Moreover, the CR values of Pb were highly correlated to SA in adults (0.66) and children (0.99). These results are the same as those in previous studies, where ED, SA and chemical concentration were significantly correlated to the non-carcinogenic and carcinogenic risks from chemical exposure [26,59].

Indonesia has limited laws regarding environmental protection, and there are no new rules on quality standards with respect to air pollution control [60]. The applicable rules are based on PP No. 41 1999 and PP. No.22 2021 [44,61]. Only TSP, Pb, NO_2_, CO_2_ and a few other pollutants have specific concentration limits that apply nationally. The laws must be enforced as the rise in industrial activity and the population growth in Indonesia could threaten the quality of the environment. The government should encourage public awareness and enforce the rules to protect the environment and the health of the general population.

## 4. Conclusions

Industrial activities play an important role in releasing total particulate matter containing PTEs into the environment. In this study, it was found that the total particulate matter (As, Cr, Cu, Pb and Zn) increased to higher levels in the dry season than in the wet season. Despite experiencing a drastic increase in concentration through seasonal changes, the total suspended particulate levels are still in accordance with the daily permissible limit set by the WHO and Indonesian guidelines. According to the Monte Carlo simulation, the probability of cancer risk from Pb and Cr for the 5% percentile and the 95% percentile is above the permissible level recommended by the US EPA, whereas the cancer risk from As is still negligible. Therefore, the impact of a long exposure duration and the toxicity of PTEs in total particulate matter should be brought to the attention of the public to help prevent adverse health effects. The findings from this study should be of critical interest to local authorities and should encourage them to pay more attention to environmental management.

## Figures and Tables

**Table 1:** Definitions, units, symbols and values for the health risk assessment.

Definitions	Units	Symbols	Values	Sources
Metal concentration	mg/m^3^ for ADD_inhalation_, and mg.kg^−1^ for ADD_oral_ and ADD_dermal_	C		Site-specific
Ingestion rate	mg.day^−1^	${\mathrm{Ing}}_{\mathrm{rate}}$	200, children 100, adults	[5,28]
Inhalation rate	m^3^.day^−1^	${\mathrm{Inh}}_{\mathrm{rate}}$	7.6, children 20, adults	[29]
Exposure duration	year	ED		Site-specific
Exposure frequency	days/year	EF	350	[29]
Conversion factor	kg.mg^−1^	CF	1 × 10^−6^	[28]
Body weight	kg	BW		Site-specific
Averaging time	days	AT	ED × 365 days	Site-specific
Particulate emission factor	m^3^/kg	PEF	1.36 × 10^9^	[29]
Dermal absorption factor		ABS	0.001	[28,29]
Skin surface area	cm^2^	SA		Site-specific
Skin adherence factor	mg.cm^−2^ h	SL	0.07, adults 0.2, children	[29]

**Table 2:** References doses (RfD), reference concentration (RfC) and cancer slope factor (CSF) from seven metals.

Metals	Dermal RfD	Ingestion RfD	Inhalation RfC	Ingestion CSF	Dermal CSF	Inhalation CSF	Sources
Al	1.00 × 10^−1^	4.00 × 10^−4^	5.00 × 10^−3^	-	-	-	[30,31]
As	3.00 × 10^−4^	3.00 × 10^−4^	3.00 × 10^−4^	1.50 × 10^−3^	1.5 × 10^−3^	1.5 × 10^−3^	[32]
Cr	6.00 × 10^−5^	3.00 × 10^−3^	8.00 × 10^−6^	5.00 × 10^−1^	4.2 × 10^−1^	41	[33,34]
Cu	12	40	1.20 × 10^−4^	-	-	-	[31,35]
Ni	5.4	20	1.40 × 10^−2^	-	-	-	[31,34]
Pb	5.20 × 10^−4^	3.50 × 10^−3^	5.20 × 10^−4^	8.50 × 10^−3^	4.2 × 10^−1^	4.2 × 10^−2^	[34,36]
Zn	60	3.00 × 10^−1^	3.60 × 10^−4^	-	-	-	[37,38]

**Table 3:** Metal concentrations in total suspended particulate (μg/m^3^) in the atmosphere compared to Indonesian and WHO standards.

		TSP	Al	As	Cr	Cu	Ni	Pb	Zn
Wet season	Aver	51.75	2558.5	1.61	11.88	4.68	1.80	6.90	9844.5
	Min	6.39	6098.8	1.45	10.71	2.72	1.44	5.54	7956.7
	Max	133.24	11,678.8	2.08	13.95	7.04	2.13	8.11	14,632.5
	Std	43.8	2558.5	0.24	1.25	1.64	0.30	0.90	2587.8
Dry season	Aver	156.86	844.50	91.63	81.17	78.97	BDL	746.78	23247.1
	Min	8.07	58.6	51.94	8.21	4.74	BDL	130.33	15,139.4
	Max	94.24	1446.66	136.81	187.8	243.7	BDL	1968.1	30,600.1
	Std	33.9	33.63	27.93	81.46	99.07	BDL	918.8	7468.7
Standard									
Indonesian [44]	230 μg/m^3^ (24 h) 90 μg/m^3^ (annually)		-	-	-	-	-	2 μg/m^3^ (24 h) 1 μg/m^3^ (annually)	-
WHO [45]	150–230 μg/m^3^ (24 h) 60–90 μg/m^3^ (annually)		-	6.6 ng/m^3^	0.001 μg/m^3^	-	25 ng/m^3^	0.5 μg/m^3^	-

BDL: below detection limit.

**Table 4:** Hazard quotient (HQ) and hazard index (HI) values from inhalation, ingestion and dermal route.

		Al	As	Cr	Cu	Ni	Pb	Zn	HI
Adults	HQInh	3.88 × 10^−4^	3.55 × 10^−4^	1.32 × 10^−2^	7.96 × 10^−4^	2.93 × 10^−7^	3.61 × 10^−8^	1.05 × 10^−1^	0.64
	HQIng	1.27 × 10^−3^	2.41 × 10^−1^	2.40 × 10^−2^	1.62 × 10^−6^	1.39 × 10^−7^	1.67 × 10^−1^	8.56 × 10^−2^	
	HQDerm	5.85 × 10^−5^	4.33 × 10^−10^	4.80 × 10^−3^	2.16 × 10^−8^	2.06 × 10^−9^	4.49 × 10^−3^	1.70 × 10^−6^	
Children	HQInh	2.93 × 10^−4^	2.61 × 10^−4^	9.78 × 10^−3^	5.86 × 10^−4^	2.16 × 10^−7^	2.66 × 10^−8^	7.73 × 10^−2^	2.12
	HQIng	4.94 × 10^−3^	9.36 × 10^−1^	9.34 × 10^−2^	6.29 × 10^−6^	5.42 × 10^−7^	6.48 × 10^−1^	3.32 × 10^−1^	
	HQDerm	1.41 × 10^−4^	2.62 × 10^−3^	1.01 × 10^−2^	5.87 × 10^−8^	5.62 × 10^−9^	1.22 × 10^−2^	4.65 × 10^−6^	

**Table 5:** Carcinogenic risk (CR) and total carcinogenic risk (TCR) for adults and children.

	Routes	As	Pb	Cr	TCR
Adults	CRInh	1.55 × 10^−10^	3.52 × 10^−8^	4.24 × 10^−8^	3.11 × 10^−5^
	CRIng	1.05 × 10^−7^	2.08 × 10^−6^	2.89 × 10^−5^	
	CRDerm	2.41 × 10^−11^	5.46 × 10^−8^	6.58 × 10^−9^	
Children	CRInh	1.14 × 10^−10^	2.60 × 10^−8^	3.14 × 10^−8^	1.32 × 10^−4^
	CRIng	4.11 × 10^−7^	1.89 × 10^−5^	1.12 × 10^−4^	
	CRDerm	1.66 × 10^−10^	3.77 × 10^−7^	4.55 × 10^−8^	

## Data Availability

The data that support the findings of this study are available on request from the corresponding author.

## Supplementary Materials

The following are available online at https://www.mdpi.com/article/10.3390/toxics9120328/s1, Figure S1: Distribution frequency of cancer risks (As, Cr and Pb) for adult and children, Table S1: Average daily dose (ADD) values from inhalation, ingestion and dermal routes.
