# Supplementary material for: Potentially Toxic Element Levels in Atmospheric Particulates and Health Risk Estimation around Industrial Areas of Maros, Indonesia
Source: Toxics. 2021 Dec 2;9(12):328. doi: 10.3390/toxics9120328 (PMC8708896; doi:10.3390/toxics9120328)
Supplement: Supplementary file 1 [file toxics-09-00328-s001.zip › toxics-1457925-supplementary.pdf]

toxics

MDPI

Supplementary Materials: Potentially Toxic Element Levels in Atmospheric Particulates and Health Risk Estimation Around Industrial Areas of Maros, Indonesia

Annisa Utami Rauf, Anwar Mallongi, Kiyoung Lee, Anwar Daud, Muhammad Hatta, Wesam Al Madhoun, Ratna Dwi Puji Astuti

Table S1. Average daily dose (ADD) values from inhalation, ingestion and dermal routes.

		Al	As	Cr	Cu	Ni	Pb	Zn
Adult	ADDInh	1.94×10^{-6}	1.06×10^{-7}	1.06×10^{-7}	9.55×10^{-8}	4.11×10^{-9}	1.65×10^{-3}	3.78×10^{-5}
	ADDIng	1.27×10^{-3}	7.24×10^{-5}	7.22×10^{-5}	6.49×10^{-5}	2.79×10^{-6}	5.85×10^{-4}	2.57×10^{-2}
	ADDDerm	5.21×10^{-6}	9.63×10^{-4}	2.88×10^{-7}	2.59×10^{-7}	1.11×10^{-8}	2.33×10^{-6}	1.02×10^{-4}
Children	ADDInh	1.46×10^{-6}	7.84×10^{-8}	7.83×10^{-8}	7.03×10^{-8}	3.02×10^{-9}	1.21×10^{-3}	2.78×10^{-5}
	ADDIng	4.94×10^{-3}	2.80×10^{-4}	2.80×10^{-4}	2.51×10^{-4}	1.08×10^{-5}	2.26×10^{-3}	9.96×10^{-2}
	ADDDerm	1.41×10^{-5}	7.86×10^{-7}	7.84×10^{-7}	7.05×10^{-7}	3.03×10^{-8}	6.35×10^{-6}	2.79×10^{-4}

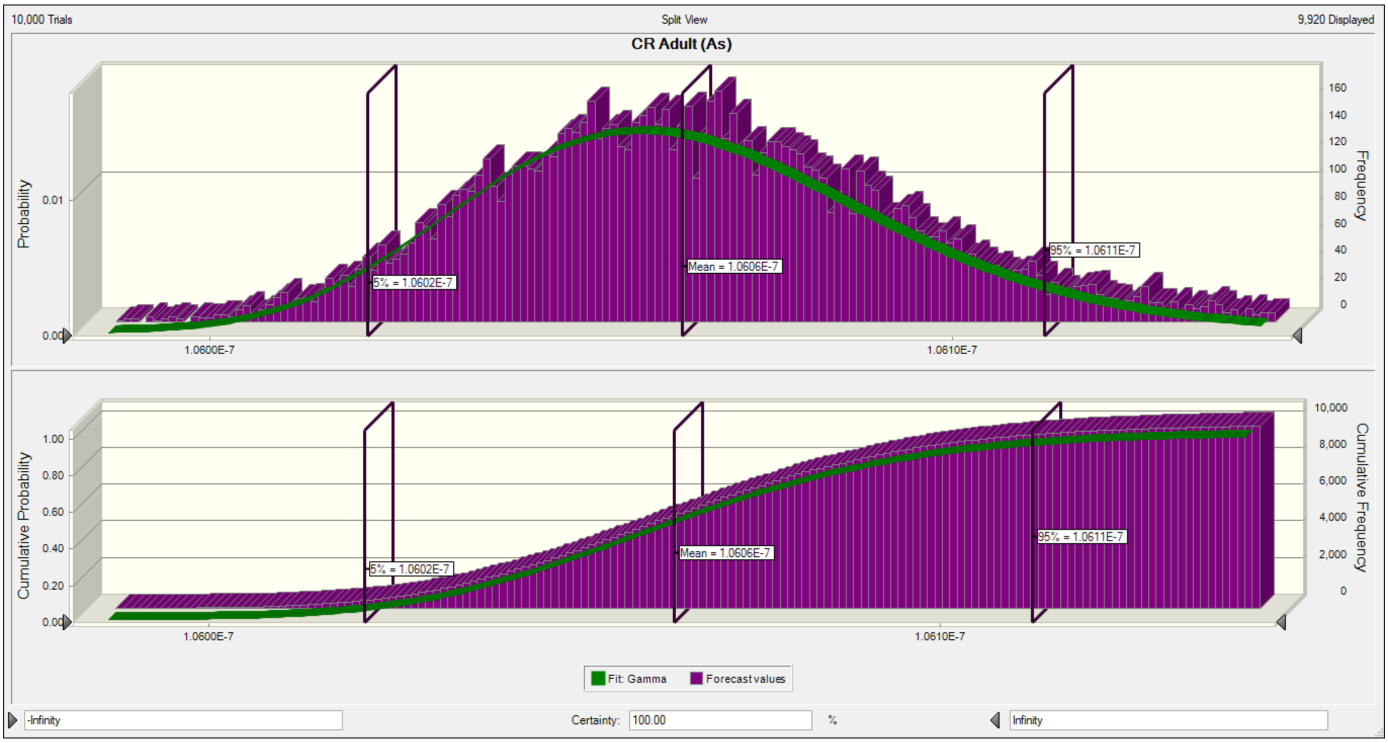

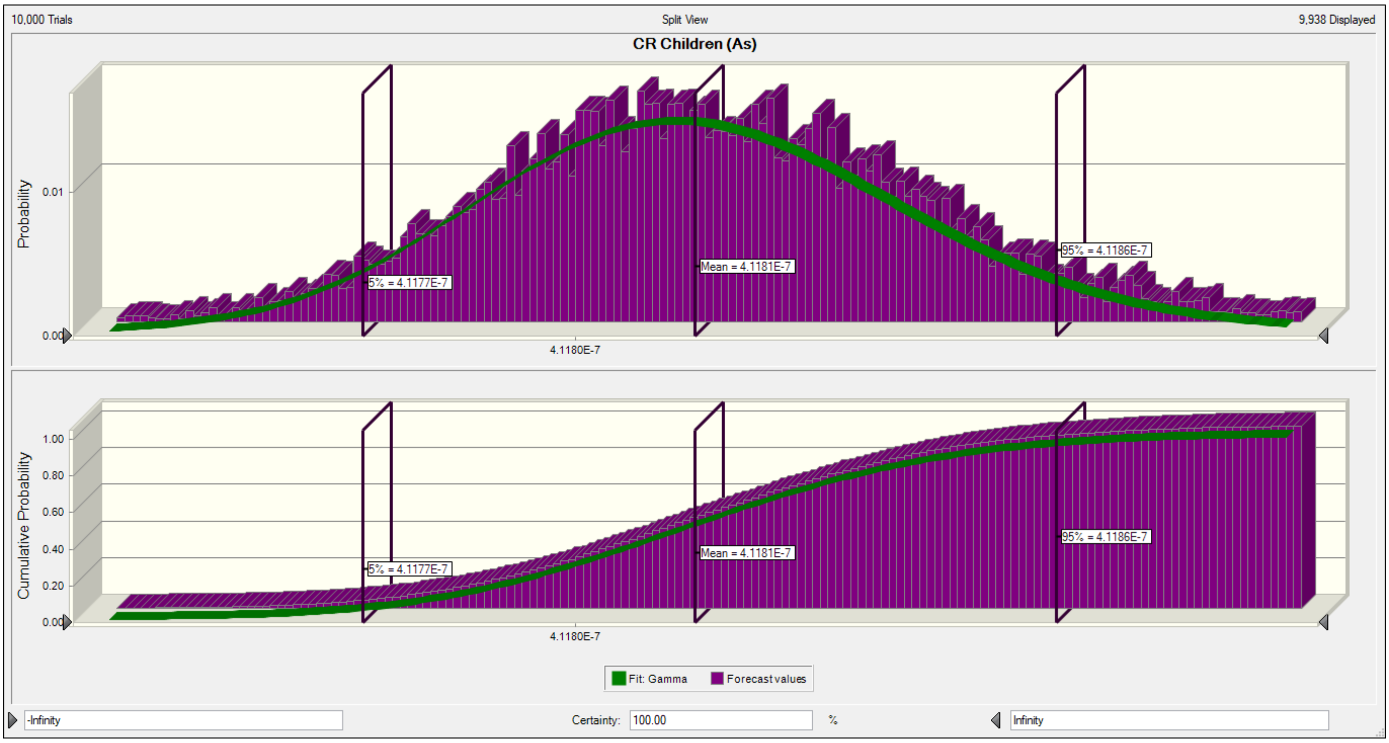

10,000 Trials
Split View
9,938 Displayed
CR Children (As)
Probability
0.01
0.00
5% = 4.1177E-7
Mean = 4.1181E-7
95% = 4.1186E-7
4.1180E-7
Cumulative Probability
1.00
0.80
0.60
0.40
0.20
0.00
Fit: Gamma
Forecast values
-Infinity
Certainty:
100.00
%
Infinity

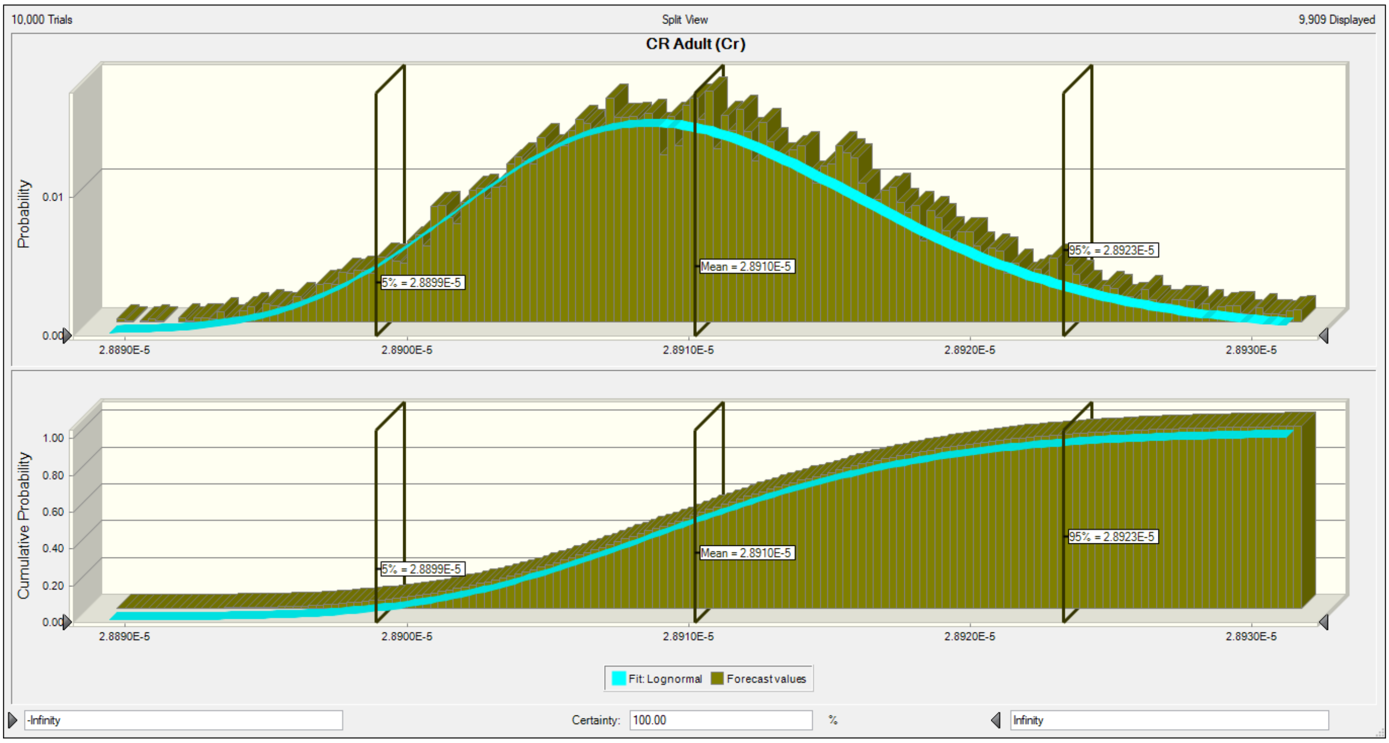

10,000 Trials
Split View
9,909 Displayed
CR Adult (Cr)
Probability
0.01
0.00
5% = 2.8899E-5
Mean = 2.8910E-5
95% = 2.8923E-5
2.8890E-5
2.8900E-5
2.8910E-5
2.8920E-5
2.8930E-5
Cumulative Probability
1.00
0.80
0.60
0.40
0.20
0.00
Fit: Lognormal
Forecast values
-Infinity
Certainty:
100.00
%
Infinity

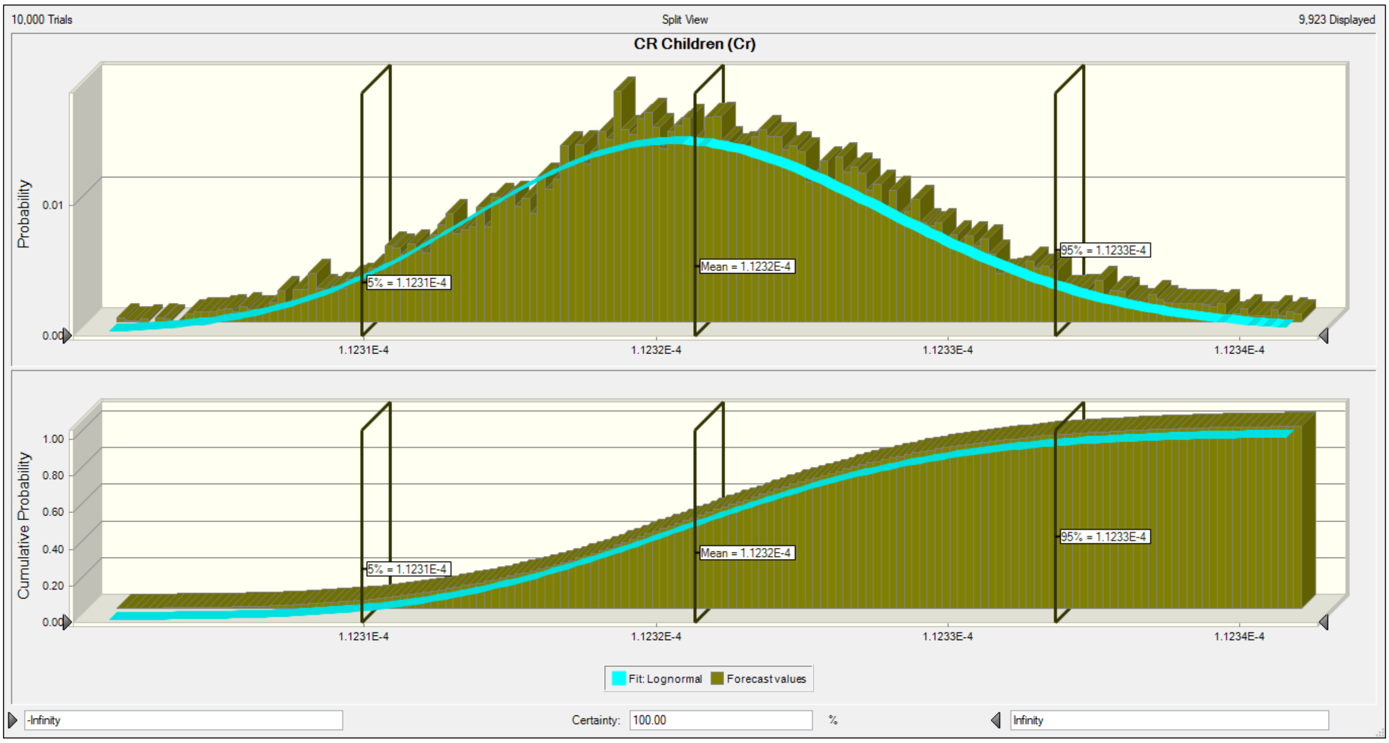
10,000 Trials
Split View
9,923 Displayed
CR Children (Cr)
Probability
0.01
0.00
5% = 1.1231E-4
Mean = 1.1232E-4
95% = 1.1233E-4
1.1231E-4
1.1232E-4
1.1233E-4
1.1234E-4
Cumulative Probability
1.00
0.80
0.60
0.40
0.20
0.00
Fit: Lognormal
Forecast values
-Infinity
Certainty:
100.00
%
Infinity

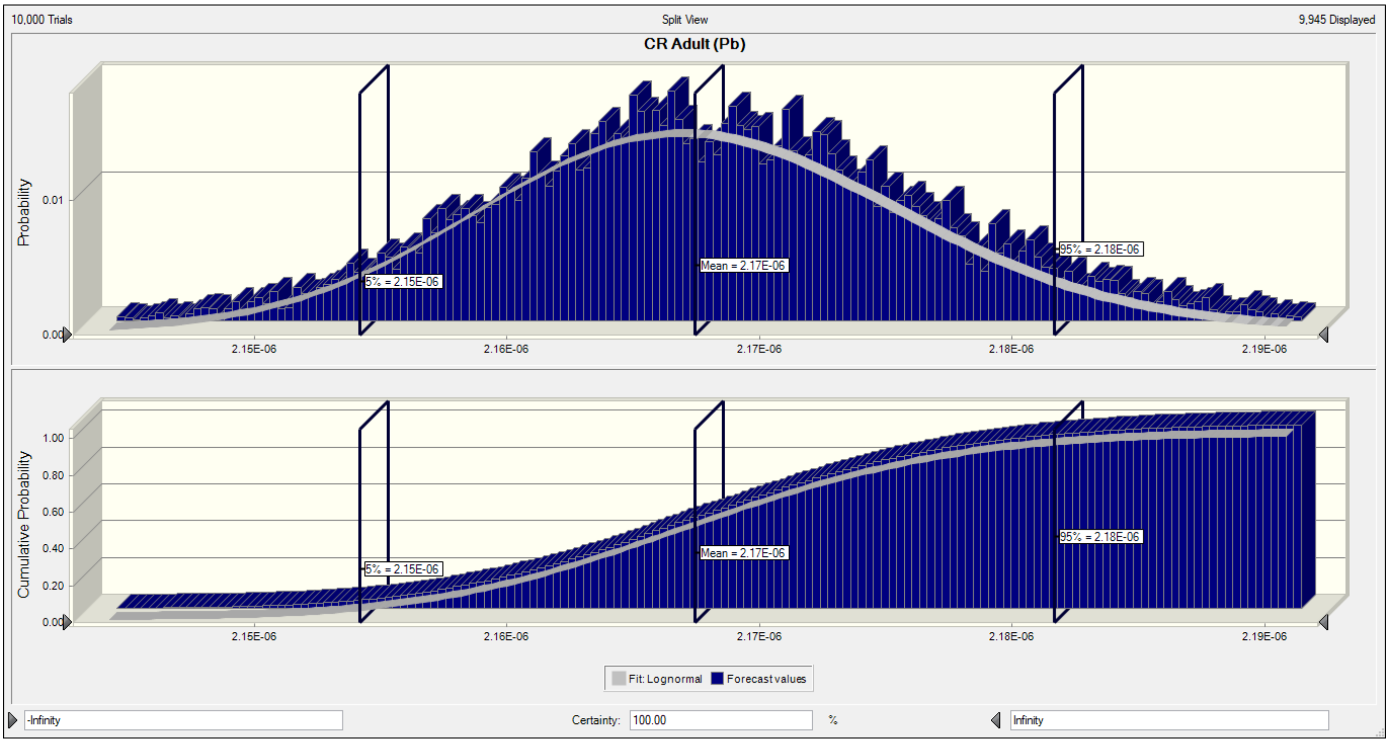
10,000 Trials
Split View
9,945 Displayed
CR Adult (Pb)
Probability
0.01
0.00
5% = 2.15E-06
Mean = 2.17E-06
95% = 2.18E-06
2.15E-06
2.16E-06
2.17E-06
2.18E-06
2.19E-06
Cumulative Probability
1.00
0.80
0.60
0.40
0.20
0.00
Fit: Lognormal
Forecast values
-Infinity
Certainty:
100.00
%
Infinity

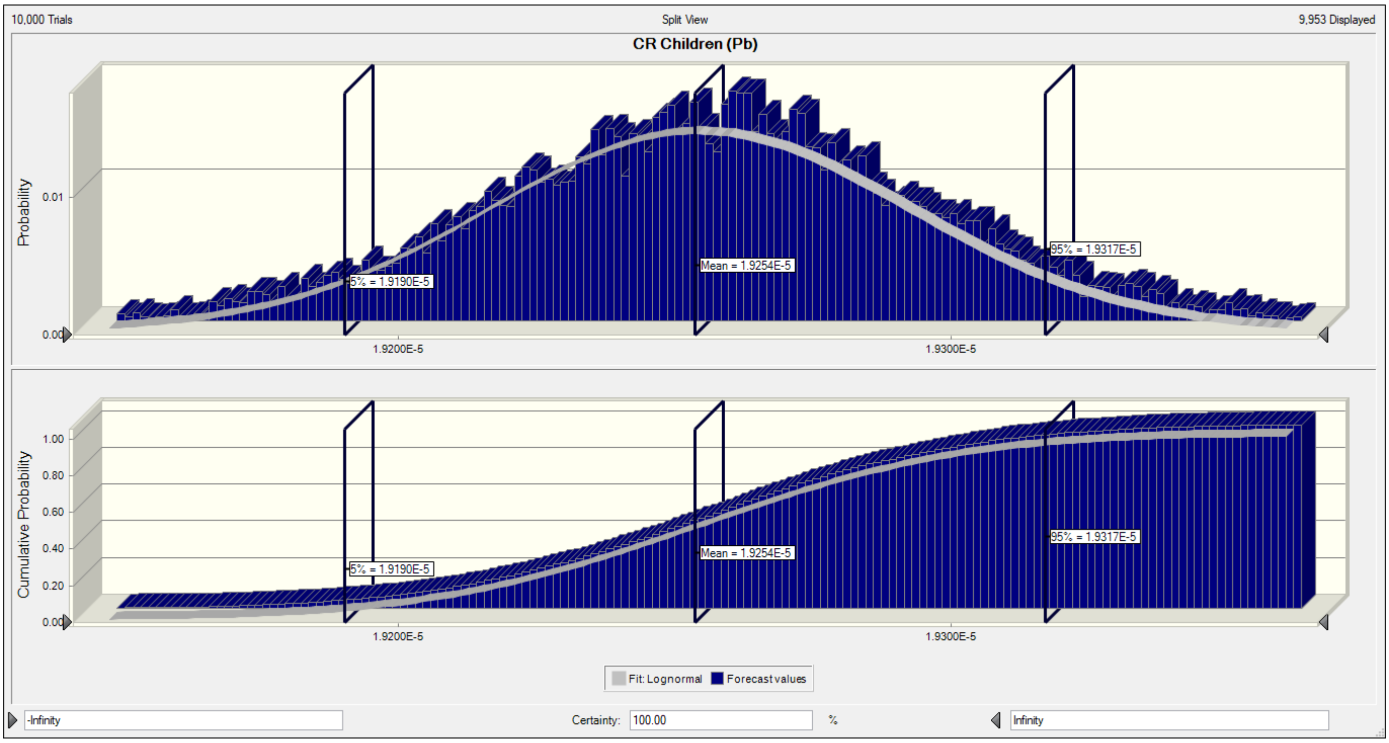

Figure S1. Distribution frequency of cancer risks (As, Cr and Pb) for adult and children.
